# Effect of GLP-1 Receptor Agonists on Renal Functions and Diabetic Nephropathy in Type 2 Diabetes Mellitus (T2DM) Patients: A Systematic Review and Meta-Analysis

**DOI:** 10.7759/cureus.71739

**Published:** 2024-10-17

**Authors:** Ali J Mohamed, Ali H AlSaffar, Ali A Mohamed, Mohamed H Khamis, Ahmed A Khalaf, Husain J AlAradi, Abdulla I Abuhamaid, Ali H Sanad, Hasan L Abbas, Abdulla M Abdulla, Osama A Alkhamis

**Affiliations:** 1 Nephrology, Eastern Health Cluster, Dammam, SAU; 2 Internal Medicine, King Saud University Medical City, King Khalid University Hospital, Riyadh, SAU; 3 Internal Medicine, College of Medicine, Mansoura University, Mansoura, EGY; 4 Emergency Medicine, Eastern Health Cluster, Dammam, SAU; 5 Internal Medicine, Eastern Health Cluster, Dammam, SAU; 6 Internal Medicine, Salmaniya Medical Complex, Manama, BHR; 7 General Practice, Al-Nabaa Medical Center, A'ali, BHR; 8 Emergency Medicine, Salmaniya Medical Complex, Manama, BHR

**Keywords:** chronic kidney disease (ckd), ckd, diabetes mellitus type 2, diabetic nephropathy, end stage kidney disease (eskd), glp1 receptor agonist

## Abstract

Diabetes mellitus (DM) causes multiple kidney problems ultimately leading to renal failure, with a marked rise in the number of patients worldwide requiring renal replacement therapy (RRT). Diabetic kidney disease (DKD) remains a leading cause of morbidity and death despite advancements in treatment; however, recent cardiovascular outcome trials have highlighted the potential benefits of glucagon-like peptide-1 (GLP-1) receptor agonists and sodium-glucose cotransporter 2 (SGLT2) inhibitors in managing chronic kidney disease (CKD) and cardiovascular risks in type 2 diabetes (T2DM) patients, leading to recommendations for their use following metformin in clinical guidelines. The meta-analysis was run on RevMan 5.4 (The Cochrane Collaboration, 2020). Risk of bias was done using the Cochrane Risk of Bias (RoB) 2 tool for the quality assessment of studies. Eleven studies were selected for this systematic review, all of which provided sufficient data for the outcomes. The effect size calculated for urinary albumin excretion rate (UAER) was calculated to be d = -0.48, CI = 95% (-1.72, 0.75) and for estimated glomerular filtration rate (eGFR) % and eGFR mL/min, it was found to be d = -0.71, CI = 95% (-2.00, 0.58) and d = -0.71, CI = 95% (-2.00, 0.58), respectively. Overall, this meta-analysis supports the use of GLP-1 receptor agonists as an effective therapeutic option to protect renal function in T2DM patients, particularly those at high risk of or with existing DKD.

## Introduction and background

Diabetes mellitus (DM) continues to be the most common cause of end-stage renal disease (ESRD) and chronic kidney disease (CKD), with a steadily growing global patient population [1]. Diabetes raises the risk of unfavorable cardiovascular outcomes in comparison to people without the illness. With a nearly three-fold higher risk of dying from coronary heart disease than people without diabetes, people with diabetes continue to have the highest death rate from cardiovascular disease (CVD) [2]. Additionally, kidney failure is more common in people with diabetes; approximately 40% of them develop CKD. The prevalence of CVD in adults 65 years of age and older with CKD is almost two-thirds higher than that of their counterparts without CKD [3]. The number of new patients with ESRD has increased significantly in 2019 [4]. Furthermore, from 19.5% in 1992 to 50.6% in 2012, the proportion of patients with DM as the primary cause of ESRD increased, making DM the most common cause of ESRD [5]. Renal replacement therapy (RRT) is predicted to be more than twice as popular in 2030 as it was in 2010, despite improvements in medical technology and treatment [6]. 

The most common cause of morbidity and death in people with diabetes is diabetic kidney disease (DKD) [7,8]. Thus, it is essential to stop DKD from starting and spreading, in part by creating effective treatment plans. The current treatment of DKD primarily uses angiotensin-converting enzyme inhibitors (ACEis) or angiotensin receptor blockers (ARBs) to control blood pressure [9]. There are currently no widely used medications or treatments intended to halt the progression of DKD [10]. Both glucagon-like peptide-1 (GLP-1) receptor agonists and sodium-glucose cotransporter 2 (SGLT2) inhibitors have demonstrated improvements in cardiorenal outcomes in several cardiovascular outcome trials (CVOTs), especially in patients with type 2 diabetes (T2DM) who have a high risk of CVD [11, 12]. After metformin in the glucose-lowering treatment plan for patients with T2DM and CKD, both the American Diabetes Association and the Korean Diabetes Association advise clinicians to consider incorporating SGLT2 inhibitors or GLP-1 receptor agonists. This guidance is supported by findings from clinical trials [13, 14].

Objectives

The objective is to evaluate the impact of GLP-1 receptor agonists on diabetic nephropathy and renal function in individuals with T2DM. The study's specific objective is to evaluate the effect of GLP-1 agonists on important renal outcomes, such as microalbuminuria, estimated glomerular filtration rate (eGFR), and urinary albumin excretion rate (UAER).

## Review

Methodology

Study Design 

For this study, a systematic review and meta-analysis were carried out. 

Eligibility Criteria

Inclusion criteria: The study population included adults aged 18 years and older diagnosed with T2DM, specifically those at risk of or with diabetic nephropathy. Comparisons included placebo, standard care, or any other antidiabetic treatment not involving GLP-1 receptor agonists, as well as studies comparing different GLP-1 receptor agonists or doses. Eligible outcomes included renal function measures, specifically UAER, eGFR, and microalbuminuria or proteinuria levels. Studies must report quantitative data or provide sufficient statistical information to calculate effect sizes for these outcomes. The study design included randomized controlled trials (RCTs), cohort studies, and controlled clinical trials, while systematic reviews and meta-analyses may be considered for reference purposes only. Studies written in English were included, with no initial restrictions on the publication date, although emphasis was placed on recent and relevant studies.

Exclusion criteria: Studies involving populations other than adults with T2DM, such as those with type 1 diabetes or gestational diabetes, were excluded. Studies that did not use GLP-1 receptor agonists as the primary intervention or those using other diabetes medications without GLP-1 receptor agonists were excluded. Studies lacking relevant renal outcome measures or those that did not provide sufficient data for effect size calculation were not included. Animal studies, in vitro studies, editorials, commentaries, reviews, conference abstracts, and studies not published in peer-reviewed journals were excluded. Studies published in languages other than English or those without full-text availability were also excluded. Additionally, studies with overlapping or duplicate data were excluded unless they provided unique data relevant to the analysis.

Search Strategy

The systematic review employed a broad search strategy to locate literature across multiple databases, including PubMed and Google Scholar. Preferred Reporting Items for Systematic Reviews and Meta-Analyses (PRISMA) guidelines were adhered to during the article search process. There were various full-text articles, abstracts, and journal titles. The search strategy made use of the Boolean operators AND/OR. To further refine the article search, more filters were suggested.

Data extraction

The search strategy included ("GLP-1 receptor agonist" OR "glucagon-like peptide-1 receptor agonist" OR liraglutide OR dulaglutide OR exenatide OR semaglutide OR albiglutide OR lixisenatide) AND ("type 2 diabetes" OR "type 2 diabetes mellitus" OR T2DM) AND ("renal function" OR "kidney function" OR "diabetic nephropathy" OR "chronic kidney disease" OR CKD OR "urinary albumin excretion rate" OR UAER OR "estimated glomerular filtration rate" OR eGFR OR microalbuminuria OR proteinuria). 

Selection Process

We looked through peer-reviewed journals and publications for pertinent literature in order to meet the inclusion criteria. Predefined inclusion and exclusion criteria were used to determine which studies were included or excluded. In the end, the review and analysis comprised eighteen studies in total. Research that failed to satisfy the eligibility requirements during the screening process was labeled as "disputed" or "excluded." There was a clear definition of the reasoning before exclusion. Studies that satisfied one or more of the following criteria were not accepted: (1) poor study design for our analysis; (2) high risk of bias; (3) inaccurate outcome measures; or (4) population-related problems. In some cases, multiple exclusion factors contributed to the rejection [15].

Statistical Analysis

Data for every variable were manually extracted for the meta-analysis. The initial method for conducting crossover studies was to compare the individual measurement differences between the intervention and control groups using data from paired t-tests. Unfortunately, these kinds of data were rarely included in the studies, so an alternative approach was taken. Statistical analysis for this study was conducted using R Studio (Posit Software, PBC, Boston, MA), focusing on data from studies without a comparator group. The analysis was primarily based on the total number of cases and associated mortality data. As the included studies did not provide comparator groups, the statistical evaluation centered on descriptive statistics and summary measures related to the incidence of John Cunningham (JC) virus reactivation and progressive multifocal leukoencephalopathy (PML) among patients treated with rituximab. This approach facilitated an examination of the overall prevalence and mortality associated with these conditions in the context of long-term rituximab therapy. Additionally, when data were presented graphically (e.g., in figures), an attempt was made to estimate numerical values for the results. A cutoff point of P ≤ 0.1 was utilized to evaluate the existence of heterogeneity, signifying genuine differences in effect sizes. Heterogeneity was used to quantify the degree of variation across studies, with a 50% threshold deemed significant.

Heterogeneity and Reporting Bias

Determining whether the variations among the studies that comprise the meta-analysis are noteworthy enough to influence the overall conclusions is known as heterogeneity assessment. To guarantee the validity and correctness of the meta-analysis's findings, this evaluation is required. The i2 and tau2 statistics can be used in addition to the R2 statistic to evaluate heterogeneity in the meta-analysis. The probability that variations in study outcomes are due to actual differences in the population under study rather than random variation can be evaluated using the Cochran Q statistic.

Quality Assessment

Systematic review: Every primary study selected for quality assessment had its bias risk assessed. A manual review was conducted of the population demographics, intervention components, and outcome measures. To conduct the quality assessment, the Cochrane Risk of Bias (RoB) 2 tool was employed.

Meta-analysis: To assess the degree of bias in the chosen studies, we conducted an online and digital resource search. Using the Cochrane criteria for bias evaluation, each primary study or RCT that satisfied the analysis requirements was evaluated independently. Random sequence generation (1), allocation concealment (2), participant and staff blinding (3), outcome assessment blinding (4), attrition bias due to inadequate outcome data (5), reporting bias, or selective reporting (6), and other biases were among the potential sources of bias. A traffic lights plot was used to graphically depict the quality assessment for each RCT. Additionally, Review Manager (RevMan version 5.4, The Cochrane Collaboration, 2020) was used to create a "forest plot" for the meta-analysis. RevMan version 3.5.1 software was used to perform a meta-analysis of the primary studies.

Synthesis Methods

For secondary screening, the Rayyan artificial intelligence (AI) tool (Rayyan Systems Inc., Cambridge, MA) was used. The title, name of the author, year, population, design of the study, sample size, intervention, comparison, and results were extracted.

Results

Data Items

**Figure 1:**
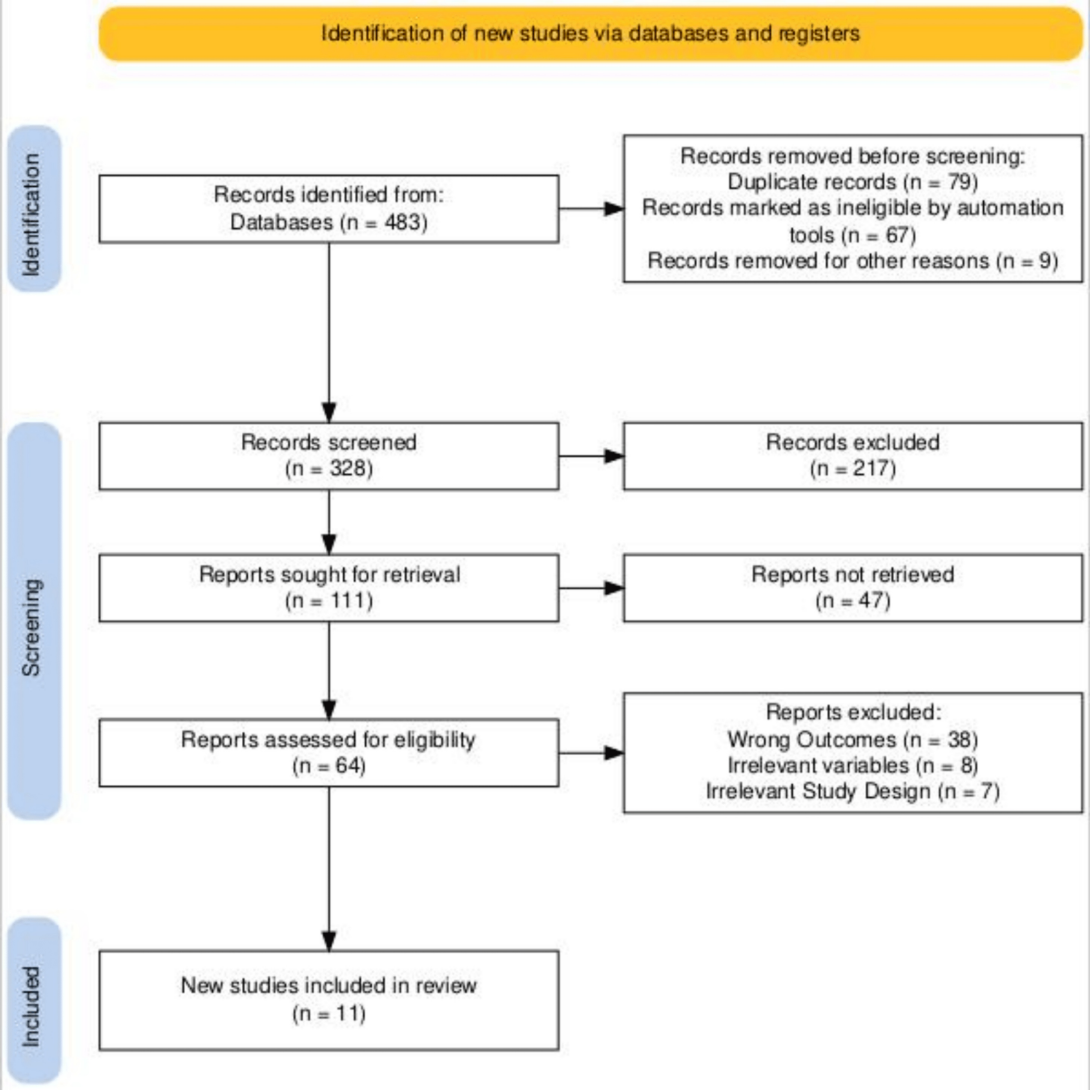
A PRISMA flow chart showcasing the selection of the included studies PRISMA: Preferred Reporting Items for Systematic Reviews and Meta-Analyses

Following the secondary screening procedure, a detailed analysis was conducted on the entire sample size (n = 11) derived from the chosen studies. To create a PRISMA flow diagram, the researchers adhered to the PRISMA guidelines. The process of selecting studies is delineated in this diagram (Figure 1), encompassing identification, screening, eligibility assessment, and incorporation of studies published in journals and other independent sources with readily available reports [16].

Study Characteristics

Eleven peer-reviewed studies made up the final sample used in the systematic analysis. A summary of the study features and demographic information from the trials that were included is shown in Table 1.

**Table 1:** Synthesis table of the included studies T2DM: type 2 diabetes mellitus; DKD: diabetic kidney disease; GLP-1: glucagon-like peptide-1; UAER: urinary albumin excretion rate; AUC GLP-1: area under the curve of glucagon-like peptide-1; DN: diabetic nephropathy; UA: uric acid; RTGC: renal triglyceride content; ePV: estimated PV; HbA1c: glycated hemoglobin; LDL: low-density lipoprotein; UACR:  urine albumin-creatinine ratio; SBP: systolic blood pressure

Sr No.	Study	Location	Study design	Sample size	Population	Intervention	Comparison	Outcomes
1	Wang et al., 2020 [17]	China	Randomized parallel study	92	T2DM patients with DKD	GLP-1 receptor agonists (exenatide)	Control groups with regular medication	Following a 24-week period, the intervention group experienced a 1.3 kg weight loss (p = 0.0001) and a significantly smaller UAER change (p = 0.0255) than the control group (2.7 kg difference). They also had lower hypoglycemia but more gastrointestinal events.
2	Johannes et al., 2017 [18]	Germany	Randomized controlled trial	9,340	Patients with type 2 diabetes	GLP-1 receptor agonists (liraglutide: incretin mimic)	Control groups with regular medication	The rates of acute kidney injury (7.1 and 6.2 events per 1000 patient-years, respectively) and other renal adverse events were comparable between the liraglutide and placebo groups (15.1 events and 16.5 events per 1000 patient-years).
3	Song et al., 2023 [19]	China	Cross-sectional study	760	Newly diagnosed T2DM patients	GLP-1 receptor agonists	Control groups with regular medication	Microalbuminuria prevalence in patients with newly diagnosed type 2 diabetes was 21.7%; this prevalence decreased across AUC GLP-1 level quartiles (27.4%, 25.3%, 18.9%, and 15.8%). After adjusting for multiple factors, patients in the highest quartile of AUC GLP-1 had a lower risk of microalbuminuria compared to those in the lowest quartile.
4	Imamura et al., 2013 [20]	Japan	Randomized controlled trial	23	Diabetic nephropathy in T2DM patients	GLP-1 receptor agonists (liraglutide: incretin mimic)	Control groups with regular medication	Liraglutide treatment for a full year led to a statistically significant reduction in proteinuria, which dropped from 2.53, 0.48 g/g creatinine to 1.47 ∡ 0.28 g/g creatinine (p = 0.002). The progression of DN was ascertained by measuring the rate of decline in the estimated glomerular filtration rate (eGFR).
5	Tonneijck et al., 2018 [21]	Netherlands	Post hoc analysis of four randomised controlled trials	132	Patients with T2DM	Immediate and prolonged GLP-1 receptor agonist	Control groups with regular medication	Exenatide infusion increases UEUA in males who are overweight and in T2DM patients; however, long-term GLP-1 receptor antagonist use has no effect on UA or UEUA in T2DM patients who have normal UA levels and low cardiovascular risk. This implies that the cardio-renal benefits of GLP-1 receptor antagonists are not mediated by UA changes.
6	Dekkers et al., 2021 [22]	Netherlands	Randomized controlled trial	50	Patients with T2DM	GLP-1 receptor agonist (liraglutide)	Control groups with regular medication	There was a significant difference in the 26-week-to-baseline RTGC ratio (95% confidence interval) between liraglutide (20.30 (20.50, 20.09)) and placebo when added to standard care.
7	Gullaksen et al.,2023 [23]	Denmark	Randomized controlled trial	65	Patients with T2DM	GLP-1 receptor agonist (semaglutide)	Control groups with empagliflozin	Mean corpus recovery (MCR) was significantly lower with semaglutide than with placebo (9%; p = 0.035), but not with empagliflozin. Empagliflozin had no effect on the urinary albumin-to-creatinine ratio (UNACR), but semaglutide reduced it by 35% (p = 0.003). The ePV fell in the combination group. There was no discernible difference in MCR between patients with and without type 2 diabetes.
8	Shaman et al., 2022 [24]	Australia	Post hoc analysis of randomized controlled trials	12637	Patients with T2DM	GLP-1 receptor agonists (semaglutide and liraglutide)	Control groups with other medication	The eGFR slope decline was significantly slowed with semaglutide 1.0 mg and liraglutide by 0.87 and 0.26 mL/min/1.73 m2 /y (P<0.0001 and P<0.001), respectively, compared with placebo. Patients with baseline eGFR <60 compared to ≥60 mL/min/1.73 showed greater effects.
9	Zobel et al., 2018 [25]	Denmark	Randomized controlled trial	279	Patients with T2DM	GLP-1 receptor agonist (liraglutide)	Control groups with other medication	Good HbA1c responders exhibited comparable changes in other risk factors in the liraglutide-treated group as compared to low responders (P ≥ 0.17). HbA1c, however, was substantially lower in respondents with good body weight than in those with low body weight (-1.6 ± 0.94% vs. -1.0 ± 0.82%, P = 0.003). Between high and low responders, changes in LDL-cholesterol, eGFR, UACR, and SBP were similar (P ≥ 0.07).
10	Jacobsen et al., 2009 [26]	New Zealand	Cohort study	30	Patients with T2DM	GLP-1 analogue (liraglutide)	Control groups with other medication	The equivalency of the area under the liraglutide AUC between groups was not observed, and no significant trend towards altered pharmacokinetics was observed among groups with escalating renal dysfunction.
11	Wajdlich et al., 2021 [27]	Poland	Crossover study	17	Patients with T2DM and impaired renal function	GLP-1 analogue (liraglutide)	Control groups with other medication	Only patients with eGFR > 60 ml/min/1.73 m2 had a decrease in systemic vascular resistance following liraglutide compared with placebo (p = 0.002), while patients with eGFR < 30 ml/min/1.73 m2 had an increase in pulse wave velocity following liraglutide compared with placebo (p = 0.0006). Only in the latter group did the 24-hour mean arterial pressure rise significantly in comparison to the placebo.

Meta-analysis

Urinary Albumin Excretion Rate

**Figure 2:**
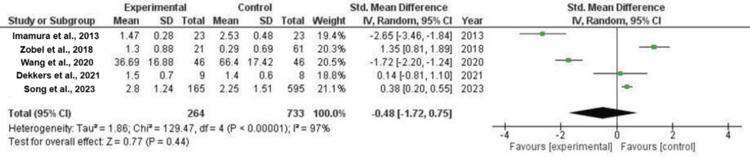
Forest plot of urine albumin excretion rate Sources: [17, 19, 20, 22, 25]

For continuous records, a forest plot was generated for each of the five-person research. A random-outcomes version was changed into selected to calculate the standardized suggested variations (SMD), in addition to the deviations and versions inside the mean (m) and standard deviation (SD). The horizontal axis displayed CI = 95%, with the plot's factor estimates represented as green squares. The vertical line inside the middle indicates "no effect." This woodland plot provided an envisioned ordinary quantitative fee for all blended results and summarized the quantitative data from every examination. After calculating the overall effect size using Cohen's d, the result was d=-0.48, CI=95% (-1.72, 0.75). The calculated values for the heterogeneity were Tau^2^ = 1.86, Chi^2^ = 129.47, df = 4 (p-value < 0.00001), and I^2^ = 97% (Figure 2).

Estimated Glomerular Filtration Rate %

**Figure 3:**
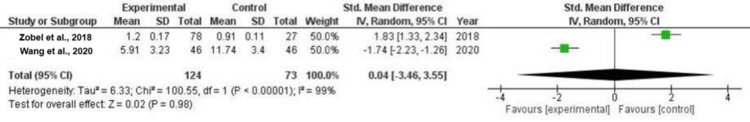
Forest plot of estimated glomerular filtration rate % Sources: [17, 25]

For two different studies, a forest plot was made for continuous data. The deviations and variations in the m, SD, and SMD were computed using a random effects model. Using Cohen's d to calculate the overall effect size, the result was d = -0.04, with a 95% CI of (-3.46, 3.55). The subsequent heterogeneity values, with one degree of freedom (df) and a p-value < 0.00001, were computed: I² = 99%; Tau² = 6.33; Chi² = 100.55. Z = 0.02 (p = 0.98) was the overall effect analysis result, meaning that every study effect individually favored the experimental group (Figure 3).

Estimated Glomerular Filtration Rate mL/min

**Figure 4:**
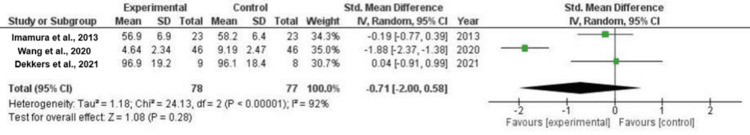
Forest plot of estimated glomerular filtration rate mL/min Sources: [17, 18, 22]

Three different studies' worth of data were combined to create the forest plot for continuous data. The SMD and deviations and variations in the m and SD were calculated using a random effects model. Using Cohen's d, the overall effect size was determined to be d = -0.71, with a 95% CI of (-2.00, 0.58). The obtained heterogeneity values were I² = 92%, Tau² = 1.18, and Chi² = 24.13, with a p-value < 0.00001 and two degrees of freedom. Z = 1.08 (p = 0.28) was the overall effect found in the analysis, meaning that all individual study effects were in favor of the experimental group (Figure 4).

Microalbuminuria

**Figure 5:**
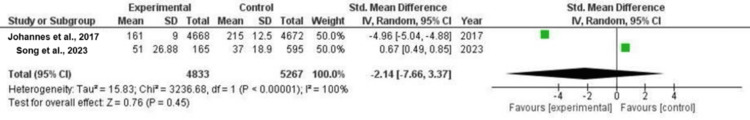
Forest plot of microalbuminuria Sources: [18, 19]

For the two distinct studies, a forest plot for continuous data was created. To compute the SMD and evaluate deviations and variations in the m and SD, a random effects model was utilized. Using Cohen's d to calculate the overall effect size, the result was d = -2.14, with a 95% confidence interval (CI) of (-7.66, 3.37). The following heterogeneity was shown by the calculations: I² = 100%, Tau² = 15.83, Chi² = 3236.68, with a p-value of 0.00001 and one degree of freedom (df). Z = 0.76 (p = 0.45) was the result of the overall effect analysis, indicating that the individual findings of each study did not demonstrate a significant influence on microalbuminuria (Figure 5).

Risk of bias in the studies

**Figure 6:**
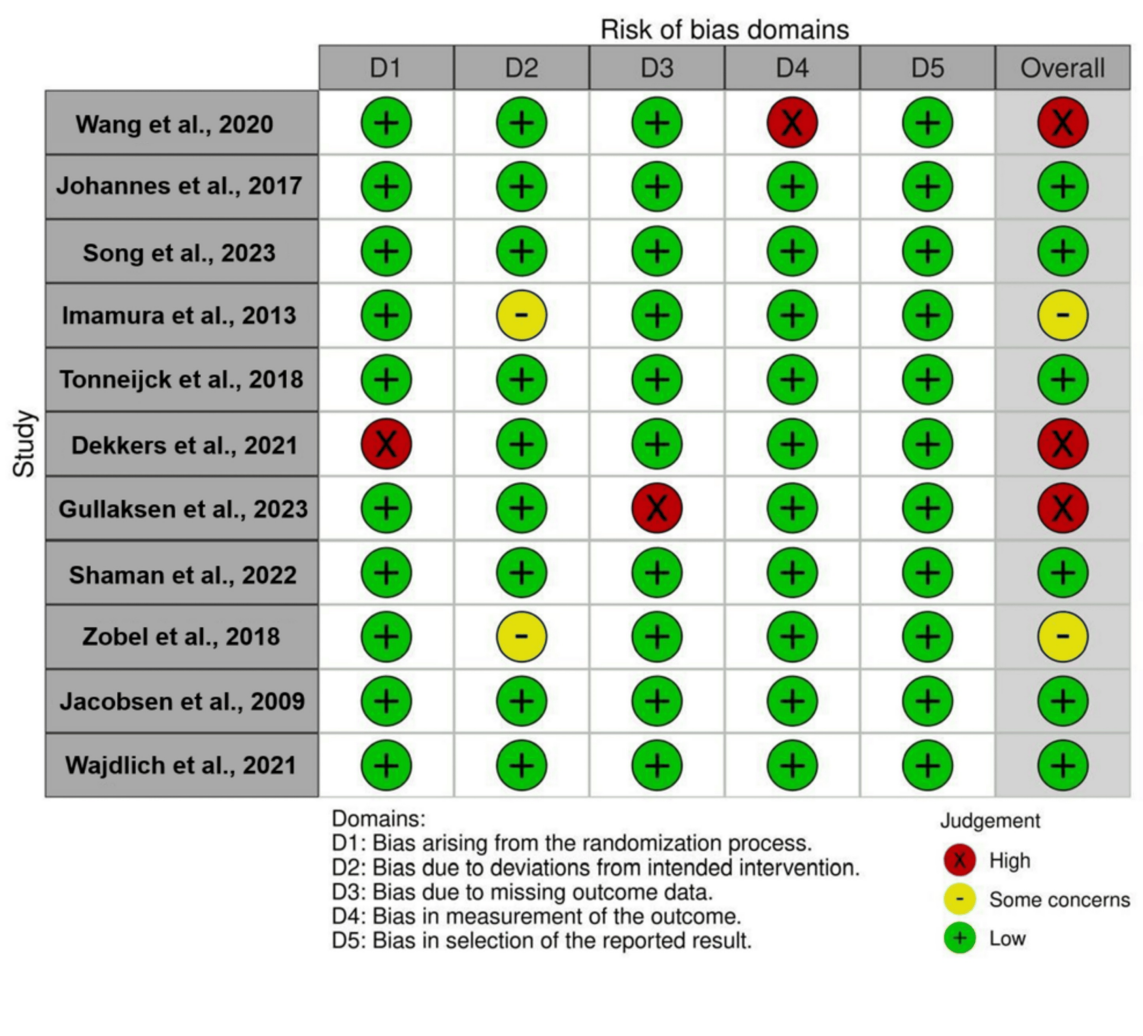
A traffic light plot to evaluate the risk of bias for the included studies Sources: [17-27]

As was previously mentioned, Cochrane ROB 2 was used to evaluate the risk of bias for each primary study that was chosen for the meta-analysis. The studies that were deemed to have an overall bias risk of "low" were then selected for the ultimate assessment. To visually represent this assessment, a traffic lights plot was created using the Cochrane Risk of Bias tool. The traffic plot for each of the six studies that were part of the analysis is shown in Figure 6 below.

Discussion

A total of 11 studies were included in this systematic review and meta-analysis. The results show that the administration of GLP-1 agonists significantly decreases the deterioration of renal function in T2DM patients. Diabetic nephropathy is shown to improve in diabetic patients after they take GLP1 agonists [17]. When administered in addition to standard care, liraglutide decreased the incidence of diabetic kidney disease development and progression when compared to placebo [18]. Postprandial GLP-1 levels were found to be independently correlated with microalbuminuria in Chinese patients with type 2 diabetes who had recently received their diagnosis [19]. One treatment option for diabetic nephropathy is liraglutide [20]. When compared to a placebo, liraglutide treatment had no effect on plasma uric acid (UA), urinary excretion of uric acid (UEUA), urinary excretion of sodium (UENa), or urine pH; in contrast, lixisenatide treatment raised UENa and urine pH from baseline but had no effect on plasma UA or urinary excretion of urea (UEU) [21]. A preliminary study indicated that 26 weeks of glycemic control led to a reduction in renal triglyceride content (RTGC), especially with liraglutide; however, larger clinical trials are necessary to confirm whether these changes genuinely reflect the impact of glycemic control on fatty kidney disease [22]. Sodium MRI, also known as 23Na MRI, can identify medication-induced changes in the mean corpus recovery (MCR) in type 2 diabetics, and semaglutide treatment for 32 weeks lowers the MCR in these patients [23]. Patients with type 2 diabetes who took semaglutide or liraglutide had kidney-protective effects; these effects were more pronounced in those who already had chronic kidney disease [24]. The response to liraglutide treatment is essentially individual; the risk factor response does not exhibit any obvious cross-dependencies, with the possible exception of the body weight and glycated hemoglobin (HbA1c) relationship [25]. This study indicates that there are no safety issues with liraglutide use in patients who have impaired renal function. Renal impairment and type 2 diabetes patients can follow standard liraglutide treatment regimens; no evidence suggests that drug exposure is increased by renal dysfunction [26]. However, heightened cardiac output linked to a sympathetic predominance and elevated heart rate may cause patients with advanced CKD receiving liraglutide to experience an increase in blood pressure. The vasodilatory effect of liraglutide is only observed to be maintained in the more advanced stages of chronic kidney disease. [27].

This systematic review and meta-analysis evaluated the impact of GLP-1 receptor agonists on renal function and diabetic nephropathy in patients with T2DM. The results indicate that GLP-1 agonists significantly slow the deterioration of renal function in T2DM patients, with beneficial effects observed in various aspects of DKD. The findings suggest that GLP-1 agonists, such as exenatide, liraglutide, and semaglutide, offer renal protection and improve diabetic nephropathy, which is crucial given the high burden of renal complications in T2DM. Despite these promising findings, some limitations need to be addressed. The heterogeneity in study design, duration of treatment, and specific GLP-1 agonists used across trials may affect the generalizability of the results. Additionally, while most studies showed beneficial effects on renal function, not all reported consistent improvements across all measured outcomes, suggesting that the impact of GLP-1 agonists may vary depending on the specific renal parameters assessed.

At present, the FLOW trial, short for Flowing Outcomes in Kidney Disease (NCT03819153), is assessing how once-weekly semaglutide affects the rate at which renal impairment advances. At the start of RRT, a sustained ≥50% reduction in eGFR, an eGFR consistently below 15 mL/min/1.73 m², and mortality from renal disease or CVD are the main renal outcomes being evaluated. With a completion date of 2024, the trial is expected to enroll more than 3,000 patients with moderate to advanced CKD, albuminuria, and T2DM. This will be the first investigation into how a GLP-1 receptor agonist affects outcomes associated with kidney disease [28]. Furthermore, the SOUL trial, short for Semaglutide cardiOvascular oUtcomes triaL (NCT03914326), an ongoing CVOT, is investigating the theory that in patients with type 2 diabetes who are at high risk for CVD, oral semaglutide lowers the risk of cardiovascular events. Renal death, initiation of RRT, a persistent ≥50% reduction in eGFR, and an eGFR consistently below 15 mL/min/1.73 m² comprise the composite renal endpoint in this trial, which is regarded as a secondary outcome. In September 2019, the Food and Drug Administration of the United States authorized oral semaglutide. GLP-1 receptor agonists are safe to use in patients with DKD who have stage 5 CKD, despite the fact that they can be given to patients with CKD and an eGFR as low as 15 mL/min/1.73 m² [29]. Further research is needed to determine this. GLP-1 receptor agonists appear to be promising treatment options for individuals with DKD, according to a study, and they have advantages over glucose-lowering medications [30]. These agents appear to have a more pronounced impact on reducing macroalbuminuria, although their influence on definitive renal outcomes, such as kidney failure or the need for RRT, remains less well-defined [30]. Patients with T2DM and CKD need to take a comprehensive approach to risk reduction because they are more vulnerable to adverse events that affect their kidneys and cardiovascular system. Angiotensin-converting enzyme inhibitors, ARBs, SGLT2 inhibitors, and the nonsteroidal mineralocorticoid receptor antagonist finerenone have all been shown to improve renal and cardiac function in patients with DKD in kidney disease outcome trials. Long-acting GLP-1 receptor agonists have shown excellent efficacy in reducing cardiovascular risks and safety profiles in T2DM patients at high cardiovascular risk, including those with DKD, in addition to their many positive characteristics. One of the renal benefits of GLP-1 receptor antagonists is reduced macroalbuminuria, which may be partly explained by the agents' capacity to lower HbA1C and blood pressure [31].

There were some potential restrictions, though. First off, there were different numbers of patients in each therapy group in the sample sizes of all included random clinical trials. Second, in some studies, the details of interventions were not given in a detailed manner. Additionally, there were some variations in intervention types among the included studies, including dosages, survival, and manner of delivery, which may have also had an impact on the results. Due to these restrictions, future studies requiring specific results will still require certain RCTs with well-designed, multicenter, and large sample numbers.

This meta-analysis generally supports the use of GLP-1 receptor agonists as an effective therapeutic option to preserve renal function in T2DM patients, particularly in those who already have or are at high risk of developing DKD. Future research should concentrate on long-term results and direct comparisons between various GLP-1 agonists in order to clarify their function in treating diabetic nephropathy and determine which patient subgroups might benefit the most. GLP-1 agonists may be a major breakthrough in the management of type 2 diabetes by lessening the burden of renal complications and, eventually, improving patient outcomes.

## Conclusions

Strong evidence is presented in this meta-analysis in favor of GLP-1 receptor agonists as a useful therapeutic option for maintaining renal function in patients with T2DM, particularly in those who are at high risk of developing DKD or who already have it. Beyond just helping to control blood sugar, GLP-1 agonists can also lower albuminuria and possibly even protect the kidneys. It is still unclear how much of an impact they have on hard renal outcomes. Long-term studies and direct comparisons between various GLP-1 agonists should be given priority in future research to determine the best way to use them in the management of diabetic nephropathy and to pinpoint patient subgroups that might benefit the most.

## References

[1] Alicic RZ, Rooney MT, Tuttle KR (2017). Diabetic kidney disease: challenges, progress, and possibilities. Clin J Am Soc Nephrol.

[2] Baena-Díez JM, Peñafiel J, Subirana I (2016). Risk of cause-specific death in individuals with diabetes: a competing risks analysis. Diabetes Care.

[3] Saran R, Robinson B, Abbott KC (2017). US Renal Data System 2016 annual data report: epidemiology of kidney disease in the United States. Am J Kidney Dis.

[4] Hong YA, Ban TH, Kang CY (2021). Trends in epidemiologic characteristics of end-stage renal disease from 2019 Korean Renal Data System (KORDS). Kidney Res Clin Pract.

[5] Jin DC, Han JS (2014). Renal replacement therapy in Korea, 2012. Kidney Res Clin Pract.

[6] Liyanage T, Ninomiya T, Jha V (2015). Worldwide access to treatment for end-stage kidney disease: a systematic review. Lancet.

[7] Li S, Wang J, Zhang B, Li X, Liu Y (2019). Diabetes mellitus and cause-specific mortality: a population-based study. Diabetes Metab J.

[8] Afkarian M, Sachs MC, Kestenbaum B, Hirsch IB, Tuttle KR, Himmelfarb J, de Boer IH (2013). Kidney disease and increased mortality risk in type 2 diabetes. J Am Soc Nephrol.

[9] Yamazaki T, Mimura I, Tanaka T, Nangaku M (2021). Treatment of diabetic kidney disease: current and future. Diabetes Metab J.

[10] Kristensen SL, Rørth R, Jhund PS (2019). Cardiovascular, mortality, and kidney outcomes with GLP-1 receptor agonists in patients with type 2 diabetes: a systematic review and meta-analysis of cardiovascular outcome trials. Lancet Diabetes Endocrinol.

[11] Neuen BL, Young T, Heerspink HJ (2019). SGLT2 inhibitors for the prevention of kidney failure in patients with type 2 diabetes: a systematic review and meta-analysis. Lancet Diabetes Endocrinol.

[12] Oh TJ, Moon JY, Hur KY (2020). Sodium-glucose cotransporter-2 inhibitor for renal function preservation in patients with type 2 diabetes mellitus: a Korean Diabetes Association and Korean Society of Nephrology consensus statement. Kidney Res Clin Pract.

[13] Hur KY, Moon MK, Park JS (2021). 2021 clinical practice guidelines for diabetes mellitus of the Korean Diabetes Association. Diabetes Metab J.

[14] American Diabetes Association (2021). 9. Pharmacologic approaches to glycemic treatment: Standards of Medical Care in Diabetes-2021. Diabetes Care.

[15] Kleinschmidt-DeMasters BK, Tyler KL (2005). Progressive multifocal leukoencephalopathy complicating treatment with natalizumab and interferon beta-1a for multiple sclerosis. N Engl J Med.

[16] Haddaway NR, Page MJ, Pritchard CC, McGuinness LA (2022). PRISMA2020: an R package and Shiny app for producing PRISMA 2020-compliant flow diagrams, with interactivity for optimised digital transparency and open synthesis. Campbell Syst Rev.

[17] Wang X, Zhang H, Zhang Q (2020). Exenatide and renal outcomes in patients with type 2 diabetes and diabetic kidney disease. Am J Nephrol.

[18] Mann JF, Ørsted DD, Brown-Frandsen K (2017). Liraglutide and renal outcomes in type 2 diabetes. N Engl J Med.

[19] Song LL, Wang N, Zhang JP, Yu LP, Chen XP, Zhang B, Yang WY (2023). Postprandial glucagon-like peptide 1 secretion is associated with urinary albumin excretion in newly diagnosed type 2 diabetes patients. World J Diabetes.

[20] Imamura S, Hirai K, Hirai A (2013). The glucagon-like peptide-1 receptor agonist, liraglutide, attenuates the progression of overt diabetic nephropathy in type 2 diabetic patients. Tohoku J Exp Med.

[21] Tonneijck L, Muskiet MH, Smits MM (2018). Effect of immediate and prolonged GLP-1 receptor agonist administration on uric acid and kidney clearance: post-hoc analyses of four clinical trials. Diabetes Obes Metab.

[22] Dekkers IA, Bizino MB, Paiman EH, Smit JW, Jazet IM, de Vries AP, Lamb HJ (2021). The effect of glycemic control on renal triglyceride content assessed by proton spectroscopy in patients with type 2 diabetes mellitus: a single-center parallel-group trial. J Ren Nutr.

[23] Gullaksen S, Vernstrøm L, Sørensen SS (2024). The effects of semaglutide, empagliflozin and their combination on the kidney sodium signal from magnetic resonance imaging: a prespecified, secondary analysis from a randomized, clinical trial. J Diabetes Complications.

[24] Shaman AM, Bain SC, Bakris GL (2022). Effect of the glucagon-like peptide-1 receptor agonists semaglutide and liraglutide on kidney outcomes in patients with type 2 diabetes: pooled analysis of SUSTAIN 6 and LEADER. Circulation.

[25] Zobel EH, von Scholten BJ, Goldman B, Persson F, Hansen TW, Rossing P (2019). Pleiotropic effects of liraglutide in patients with type 2 diabetes and moderate renal impairment: individual effects of treatment. Diabetes Obes Metab.

[26] Jacobsen LV, Hindsberger C, Robson R, Zdravkovic M (2009). Effect of renal impairment on the pharmacokinetics of the GLP-1 analogue liraglutide. Br J Clin Pharmacol.

[27] Wajdlich M, Nowicki M (2021). Hemodynamic effect of a single dose of glucagon-like peptide 1 receptor (GLP-1R) agonist liraglutide in patients with diabetic kidney disease. J Physiol Pharmacol.

[28] Perkovic V, Tuttle KR, Rossing P (2024). Effects of semaglutide on chronic kidney disease in patients with type 2 diabetes. N Engl J Med.

[29] McGuire DK, Busui RP, Deanfield J (2023). Effects of oral semaglutide on cardiovascular outcomes in individuals with type 2 diabetes and established atherosclerotic cardiovascular disease and/or chronic kidney disease: Design and baseline characteristics of SOUL, a randomized trial. Diabetes Obes Metab.

[30] Yu JH, Park SY, Lee DY, Kim NH, Seo JA (2022). GLP-1 receptor agonists in diabetic kidney disease: current evidence and future directions. Kidney Res Clin Pract.

[31] Michos ED, Bakris GL, Rodbard HW, Tuttle KR (2023). Glucagon-like peptide-1 receptor agonists in diabetic kidney disease: a review of their kidney and heart protection. Am J Prev Cardiol.

